# The miRNome of canine invasive urothelial carcinoma

**DOI:** 10.3389/fvets.2022.945638

**Published:** 2022-08-22

**Authors:** Mara S. Varvil, Taylor Bailey, Deepika Dhawan, Deborah W. Knapp, José A. Ramos-Vara, Andrea P. dos Santos

**Affiliations:** ^1^Department of Comparative Pathobiology, College of Veterinary Medicine, Purdue University, West Lafayette, IN, United States; ^2^Department of Veterinary Clinical Sciences, College of Veterinary Medicine, Purdue University, West Lafayette, IN, United States; ^3^Center for Cancer Research, Purdue University Center for Cancer Research, West Lafayette, IN, United States

**Keywords:** microRNA, urothelial carcinoma, miRNome, RAS, MAPK, DAPK, UP3KA

## Abstract

Urothelial carcinoma (UC) comprises up to 2% of all naturally occurring neoplasia in dogs and can be challenging to diagnose. MicroRNAs (miRNAs) have been reported to be dysregulated in numerous diseases, including neoplasia. MiRNA expression has been evaluated in human UC, but there is limited information regarding the miRNA transcriptome of UC in dogs. Our study aimed to evaluate differential miRNA expression in bladder tissue collected from normal canine urothelium and canine invasive UC (iUC) to elucidate the dysregulated pathways in canine UC. Next-Generation RNA sequencing (RNA-Seq) was performed for dogs with UC (*n* = 29) and normal canine urothelium (*n* = 4). Raw RNA data were subjected to normalization, and pairwise comparison was performed using EdgeR with Benjamini-Hochberg FDR multiple testing correction (*p* < 0.05; >2-fold change) comparing tissue samples of normal urothelium to canine iUC samples. Principal component analysis and hierarchical cluster analysis were performed. MiRNA of FFPE tissue samples of separate iUC (*n* = 5) and normal urothelium (*n* = 5) were used to evaluate five miRNAs using RT-qPCR. Pathway analysis was performed utilizing miRWalk, STRING database, and Metascape utilizing KEGG pathways and GO terms databases. Twenty-eight miRNAs were differentially expressed (DE) by RNA-Seq. RT-qPCR confirmed that four miRNAs are significantly downregulated in UC compared to healthy urothelial samples (miR-105a, miR-143, miR-181a, and miR-214). Principal component analysis and hierarchical cluster analysis showed separation between miRNAs in iUC and the control group. The DE miRNAs are most often associated with gene silencing by miRNA, miRNAs in cancer, and miRNAs involved in DNA damage responses. Proteins involved include HRAS, KRAS, ARAF, RAF1, MAPK1, MAP2K1, MAPK3, FGFR3, EGFR, HBEGF, RASSF1, E2F2, E2F3, ERBB2, SRC, MMP1, and UP3KA. The differential expression of miRNAs in canine iUC compared to normal canine urothelial tissue indicates that these markers should be further evaluated for their potential role as diagnostic and therapeutic targets.

## Introduction

Urothelial carcinoma (UC) comprises approximately 2% of all naturally occurring canine cancers (1), making it the most common urogenital cancer in dogs (2–4). Most UC are of the high-grade invasive form (iUC), with 78% of dogs with UC having tumors invading the bladder wall and 20% of neighboring organs (1, 2). At the time of diagnosis, approximately 20% of dogs have evidence of metastasis (3), with distant metastasis being associated with a worse prognosis (1, 2, 4). In one study, 58% of dogs were found to have distant metastasis at the time of death (1). When not controlled, the primary tumor may obstruct the urinary tract and cause death (1, 3). Canine iUC has been identified as a naturally occurring model for human muscle-invasive UC (1, 3, 4). As such, the molecular environment has been shown to be comparable between the two species (1, 3–5). There are distinct subtypes of UC identified, basal and luminal (5). Similar to humans, in dogs, the luminal subtype comprises 62% of tumors and commonly has *FGFR3, ERBB2*, and *ERBB3* activating mutations and is generally associated with a better prognosis (5). The basal subtype represents 38% of tumors, is enriched with *EGFR* and *HIF-1* expression, and is often metastatic at presentation (5). The basal form can have squamous and sarcomatoid histologic features and biomarkers associated with epithelial to mesenchymal transition, such as *MMP9, SERPINE2, CAV1, KRT14*, and *RASA3* (5).

Many canine patients with UC present with nonspecific urinary tract clinical signs, such as stranguria, pollakiuria, or hematuria (2). Finding abnormal epithelial cells in the urine along with a mass lesion or thickened bladder wall may increase the suspicion of UC; however, this in itself is not diagnostic, as other conditions may present similarly (2). Additionally, concurrent lower urinary tract disease commonly results in inconclusive results by cytological evaluation of the urine. Histologic evaluation is used for the definitive diagnosis of UC (2). The histologic evaluation of the tumor biopsy requires tissue samples collected by cystoscopy, catheter biopsy, or surgery. Surgery is an invasive procedure. Surgery and cystoscopy require general anesthesia. With cystoscopy, visual examination of the bladder is possible, along with the ability to obtain biopsies for histologic evaluation; however, cystoscopy requires specialized equipment and training that are not universally available. Obtaining an adequate tissue sample size may also be challenging since it is limited by the size of the tools inserted through the urethra. Immunohistochemistry for uroplakin II and III can be used to help distinguish UC from other poorly differentiated carcinomas (1, 2). The most common marker used is uroplakin III; however, while it is a highly specific marker, it has a relatively low sensitivity (6). Currently, CADET^Ⓡ^ BRAF mutation testing (Antech Diagnosis, Inc.) is commercially available for urine samples and is marketed to aid in UC diagnosis. The mutation of the BRAF gene results in the activation of the MAP kinase pathway, which drives aberrant cell growth, and proliferation (7, 8). Although the BRAF mutation detection test has the advantage of being a urine-based test, it has limitations. First, BRAF mutation is present in cancers other than UC, such as prostatic carcinoma (9), which can also be detected within the urinary tract. Moreover, detecting BRAF mutations does not equate to disease development (9). Most importantly, the absence of BRAF mutations cannot rule out UC as up to 20% of the cases do not have the mutation. Thus, a negative BRAF mutation test cannot rule out UC, nor can a positive test confirm UC specifically (9). While an adjunct test, CADET^Ⓡ^ BRAF-*PLUS*, has been marketed as a companion diagnostic test used to potentially identify patients that have UC that do not harbor the BRAF mutation, with reported improved sensitivity up to 95% for UC detection (10), these tests require further study, and there is still a need for identification of additional non-invasive molecular biomarkers for the diagnosis of this disease.

MicroRNAs (miRNAs) have become established biomarkers of cancer (11, 12) because of their stability and abundance in body fluids and reported alterations in miRNA expression in neoplastic diseases (13). MiRNAs are a regulatory class of RNAs first discovered in 1993 (9); they are short (18–24 nucleotides in length) non-coding RNAs responsible for post-transcriptional regulation of gene expression. It is thought that more than half of miRNA genes are located in cancer-associated genomic regions (14). Microarray expression data has shown that aberrant miRNA expression is present in cancer, including bladder, breast, colon, gastric, lung, prostate, and thyroid cancers (14). MiRNA may exhibit oncogenic function in some tumors but act as a tumor suppressor in other types of cancer (14). While aberrant miRNA expression has been documented in UC in dogs (15–17), a complete miRNA expression profile of canine iUC has not yet been identified.

This study evaluated the whole miRNA expression profile (miRNome) of canine UC compared with normal canine urothelial tissue. Our work which includes RNA sequencing (RNA-Seq) to obtain a complete list of miRNAs expressed in normal canine bladder and iUC tumor tissue, will build on earlier reports of miRNA expression in canine UC (16, 17). In addition, we have validated differentially expressed (DE) miRNAs from the RNA-Seq data by quantitative reverse transcription (RT-qPCR) in a different cohort of tissue samples.

## Materials and methods

### RNA-sequencing

#### Ethics statement

Owner consent was obtained for patients undergoing cystoscopy for diagnostic evaluation. A tissue sample from this procedure was collected and saved for RNA sequencing analysis. This study was approved by the Purdue Animal Care and Use Committee (Approval Number 1111000169).

#### Tissue collection

Treatment naïve canine iUC tissue samples (*n* = 29) were collected by cytoscopic biopsy (5). Patients presenting for cytoscopic biopsy were selected with no exclusion based on age, breed, or sex (5). Diagnosis of iUC was confirmed by histologic evaluation by a board-certified pathologist (JARV); all tumors were high grade with >95% epithelial cellularity (5, 18) (Supplementary Table 1). Normal bladder tissue samples (*n* = 4) were obtained from dogs that underwent euthanasia for other reasons than bladder-related diseases (5). The urothelial layers were dissected out of the tissues and stored in Trizol^™^ reagent (Invitrogen, Carlsbad, CA, USA) at−80°C until processing (5).

#### RNA sequencing

RNA sequencing procedures were run as previously described (5). Total RNA isolation and purification were achieved using the RNAeasy kit (QIAGEN, Valencia, CA, USA) following the manufacturer's instructions (5). Nanodrop spectrophotometer (NanoDrop Technologies, Wilmington, DE) and Agilent Bioanalyzer (Agilent Technologies, Santa Clara, CA) were used to evaluate the RNA quality, and an RNA integrity number of seven or greater was considered acceptable. Ribo-Zero rRNA removal kit (Illumina) was used to remove Ribosomal-RNA, and mRNA libraries were constructed (ScriptSeq v2 RNA-Seq library preparation kit, Epicenter Biotech, Madison, WI). Di- tagged cDNA was amplified by limit-cycle PCR and purified using AMPure XP System (Beck- man Coulter) (19, 20). Illumina HiSeq 4000 platform was used to generate paired-end 150 bp sequence reads, obtaining an average of 50 million reads/sample. Raw reads were cleaned for PCR artifacts, adapter trimmed, and then aligned to the canine genome (CanFam 3.1 reference genome) using COBWeb to obtain expression levels for annotated genes and isoforms (Strand NGS, v3.1, Build 235027, Agilent Technologies, Santa Clara, CA). RNA sequencing was performed by the Biomedical Genomics Core at Nationwide Children's Hospital, Columbus, OH, USA (5).

### Data validation

#### Tissue collection

Archived formalin-fixed, paraffin-embedded (FFPE) tissues were utilized. Histologically confirmed UC (*n* = 5) and normal urinary bladder mucosa (*n* = 5) from adult dogs were selected; BRAF status was unknown in these patients. All slides were reviewed by a board-certified pathologist (JARV).

#### Biomarker selection

Five DE miRNAs by RNA-Seq with a fold change greater than two or with a relevant role in UC, i.e., members of the miR-143/miR-145 cluster (21–25), were chosen for validation by RT-qPCR: miR-32, miR-105a, miR-143, miR-181a-1, and miR-214.

#### MiRNA extraction

An Illinois sternal bone marrow aspiration needle was utilized to acquire tissue cores. The needles were cleaned before coring the samples by submerging them in bleach, 80% ethanol, and RNase-free water. H&E stained tissue sections were evaluated, then the desired area of the tumor or normal mucosa was marked on the corresponding area of the FFPE tissue block. The Illinois sternal needle was used to take a core punch out of the marked area. The core was then placed in a 2 mL microcentrifuge tube for further processing. RNA was extracted from the tissue cores using the miRNeasy FFPE kit (QIAGEN), following the protocol starting with step four with one section per sample. The control cel-miR-39 (miRNeasy- Serum/Plasma Spike-in, QIAGEN, 3.5 μL/sample) was added to each sample after adding Buffer PKD.

#### Reverse transcription, quantitative polymerase chain reaction

Reverse transcription to cDNA was performed with miScript Reverse Transcriptase Mix (QIAGEN) using 50 ng of RNA for each reaction. Samples were stored at−20°C until qPCR. The miScript SYBR green kit (QIAGEN) was used to run qPCR on a QuantStudio 3 Real-Time PCR System (ThermoFisher Scientific, Waltham, MA). Primers assays for selected miRNAs, miR-32, miR-105a, miR-143, miR-181a-1, and miR-214, were acquired (QIAGEN). Melting curves were evaluated for each run, ensuring all reactions had a single peak on the melting curve and verifying the amplification of a unique PCR product.

The small nuclear RNU6B and three additional miRNAs, miR-152, miR-872, and miR-1842, were evaluated as endogenous reference genes for the normalization of miRNA expression by RT-qPCR. These markers were chosen based on RNA-Seq evaluation for consistent expression throughout control and iUC samples and literature review (26–29). The exogenous control miR-39 was also used.

### Statistical analysis

#### RNA sequencing

Statistical analysis was evaluated using Strand NGS (20), as previously described (5). Briefly, raw RNA-Seq data were normalized using TMM and DESeq (30), separately, filtered by read metrics, and quantified. For the TMM normalized data, EdgeR (31) was used for pairwise comparison, and DEseq2 was used on DESeq normalized data with Benjamini-Hochberg FDR multiple testing correction (32) (*P* < 0.05; >2-fold) to compare normal mucosa/urothelial layer samples vs. canine iUC samples (Supplementary Table 2). DE miRNAs identified (Table 1) were pooled and filtered by valid expression values within the standardized read count data, defined as a value above the limit of detection. Only miRNAs with at most one invalid value across all samples were retained. Missing values were imputed by the classification and regression tree (CART) method prior to downstream analysis (33, 34). Agglomerative hierarchical clustering was performed with Pearson correlation distance and average linkage (34). Principal component analysis (PCA) was performed with data scaled to unit variance (34). PCA and hierarchical clustering were performed on R (v4.1.2) with RStudio (v21.09.0).

**Table 1:** Differentially expressed miRNAs in invasive urothelial carcinoma compared to healthy bladder urothelial control tissue by RNA-Seq.

**miRNA**		**Entrez ID**	**Fold change**	***P*-value**	**Regulation**
1	cfa-miR-582	100886025	3.6026363	1.50E-04	Down
2	cfa-miR-145	100885996	3.1505983	9.79E-04	Down
3	cfa-miR-143	100885924	3.1213033	0.001847344	Down
4	cfa-miR-99a-2	100885929	1.5352738	0.029259656	Down
5	cfa-miR-429	100886110	−2.0079737	0.017569073	Up
6	cfa-miR-181a-1	100886055	4.337332	1.41E-07	Down
7	cfa-miR-214	100886064	3.5897517	1.70E-07	Down
8	cfa-miR-329b	100886070	3.6712651	0.027805874	Down
9	cfa-miR-544	100886022	4.0448046	0.004256103	Down
10	cfa-miR-301a	100886065	2.4762766	0.002809253	Down
11	cfa-miR-216a	100885988	3.983276	0.001502375	Down
12	cfa-miR-874	100886033	1.9066821	0.027094653	Down
13	cfa-miR-876	100886034	5.311574	0.003768745	Down
14	cfa-miR-32	100886112	1.8807616	0.006983339	Down
15	cfa-miR-7-3	100886063	4.316944	0.021464972	Down
16	cfa-miR-190a	100886156	1.8281796	0.00221304	Down
17	cfa-miR-802	100886032	4.990947	9.65E-06	Down
18	cfa-miR-568	100886024	1.8237189	0.004600147	Down
19	cfa-miR-551b	100886100	4.733979	0.018431272	Down
20	cfa-miR-532	100885973	3.4177957	1.77E-04	Down
21	cfa-miR-223	100886152	2.346452	0.002304758	Down
22	cfa-miR-374b	100886177	−1.0458393	0.00444675	Up
23	cfa-miR-361	100885969	2.1139793	0.017621992	Down
24	cfa-miR-652	100885964	2.9247227	0.007459959	Down
25	cfa-miR-764	100886168	4.5455575	0.029226616	Down
26	cfa-miR-450a	100886076	3.089305	0.012043307	Down
27	cfa-miR-105a	100886005	4.703663	0.004528188	Down
28	cfa-miR-490	100886003	4.7942433	0.014179528	Down

#### Reference gene identification

The miRNAs expressed in the RNA-Seq data were evaluated for a standard deviation of <0.05 (*n* = 40). Within that group, an F-test was performed with the hypothesis that the control and test groups did not show significant variation between the control and the iUC samples. Four miRNAs had a significant (<0.05) *p*-value and were excluded. A two-tailed Student's *t*-test was performed on the included 36 miRNAs, and 19 of these miRNAs were found to have a statistically significant difference between the control group and the iUC samples and were also excluded. A literature review was performed, and 15 miRNAs with known association with neoplasia were excluded, and two miRNAs previously used as reference genes, miR-152 (26) and RNUB6 (27–29), were included. NormFinder software was used to assess the stability of the potential reference genes based on quantification cycle (CQ) values (Table 2). This algorithm ranks the candidate reference genes according to their expression stability using a mathematical model of gene expression described by Lindbjerg et al. (35).

**Table 2:** Median quantification cycle (CQ), standard error calculation, and stability values of candidate reference genes calculated using the NormFinder algorithm.

**miRNA**	**Median CQ value**	**Standard error**	**Stability value**
miR-872	37.6142349	N/A^**^	6.91
miR-1842^*^	34.5058289	0.10131598	0.45
miR-152	29.6204967	0.56100288	2.464
RNU6B^*^	27.0446491	0.0991406	0.794

* Results with a stability value of <1.0 are considered acceptable for use as reference genes.

** Not enough CQ values were identified to perform this calculation.

#### RNA-Seq validation

A modified version of the ΔΔCQ method that uses two reference genes was utilized to evaluate the relative expression of each miRNA (36). Fold change was calculated, and a Student's *t*-test was used to compare gene expression between controls and iUC samples (Table 3).

**Table 3:** RT-qPCR evaluation of a selected differentially expressed miRNAs on the RNA-Seq evaluation.

**miRNA**	**Fold change**	**Student's *t*-test *p*-value**
miR-32	−1.416834	0.45852527
miR-105a^*^	−29.867963	0.006636535
miR-143^*^	−74.721068	0.03177502
miR-181a^*^	−0.3213207	0.03109635
miR-214^*^	−0.0782834	0.000031425
miR-374b^*^	−0.3352548	0.00647716

Fold change comparison and Student's t-test p-value for gene expression comparison of canine invasive urothelial carcinoma and normal urothelium.

* Indicates statistical significance with p < 0.05.

### Pathway analysis

Pathway analysis was performed using the Metascape software (37), miRWalk (38), and STRING database (39) with respect to the Kyoto Encyclopedia of Genes and Genomes (KEGG) pathways (40) and Gene Ontology (GO) terms. All database searches were performed using default filtering parameters. The Metascape analysis program identified statistically enriched terms (GO/KEGG terms), then accumulative hypergeometric *p*-values and enrichment factors were calculated and used to filter the terms. The significant terms were hierarchically clustered into a tree based on Kappa-statistical similarities among their genes. Then 0.3 kappa score was applied as the threshold to cast the tree into term clusters (37). A Gene Set Enrichment Analysis (GSEA) was performed on the *Canis lupus familiaris* bladder cancer dataset (Supplementary Table 3).

## Results

### RNA sequencing

Two hundred and eight miRNAs were found to be expressed in the canine bladder (Supplementary Table 4), with 41 miRNAs minimally expressed (<0.001 from baseline). One hundred and thirteen miRNAs were expressed in both normal control and iUC samples, 44 were found only in normal control urothelial samples, and ten were found only in iUC samples. Twenty-eight miRNAs are found to be DE in iUC samples when compared to control samples (*P* < 0.05) (Table 1).

**Figure 1:**
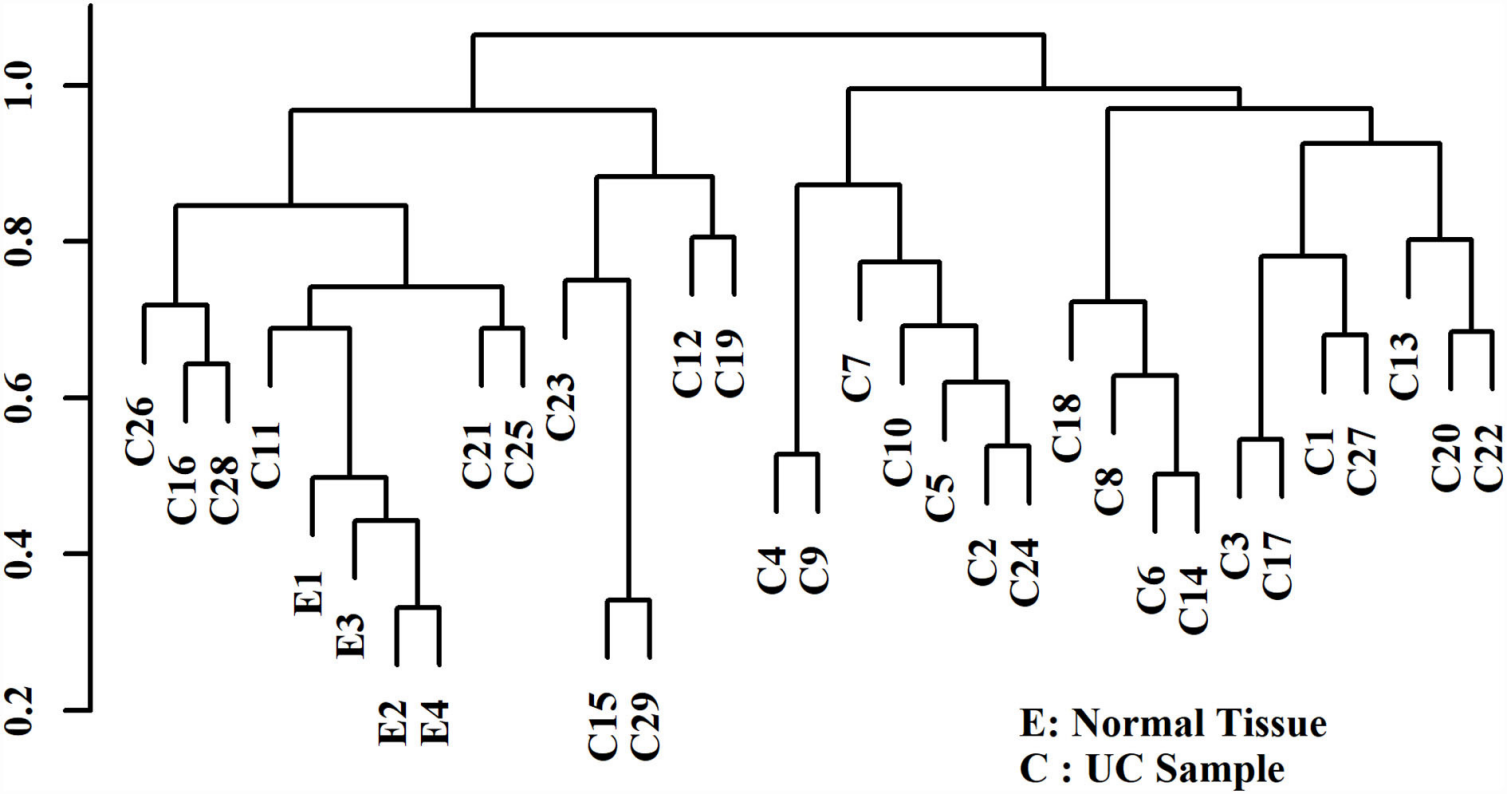
Agglomerative hierarchical clustering with Pearson's correlation distance displaying similarity of the control urothelial tissue samples (E1-4) relative to the invasive urothelial carcinoma samples (C1-29). The control urothelial tissue samples (E) demonstrate mutual similarity relative to the iUC samples (C).

**Figure 2:**
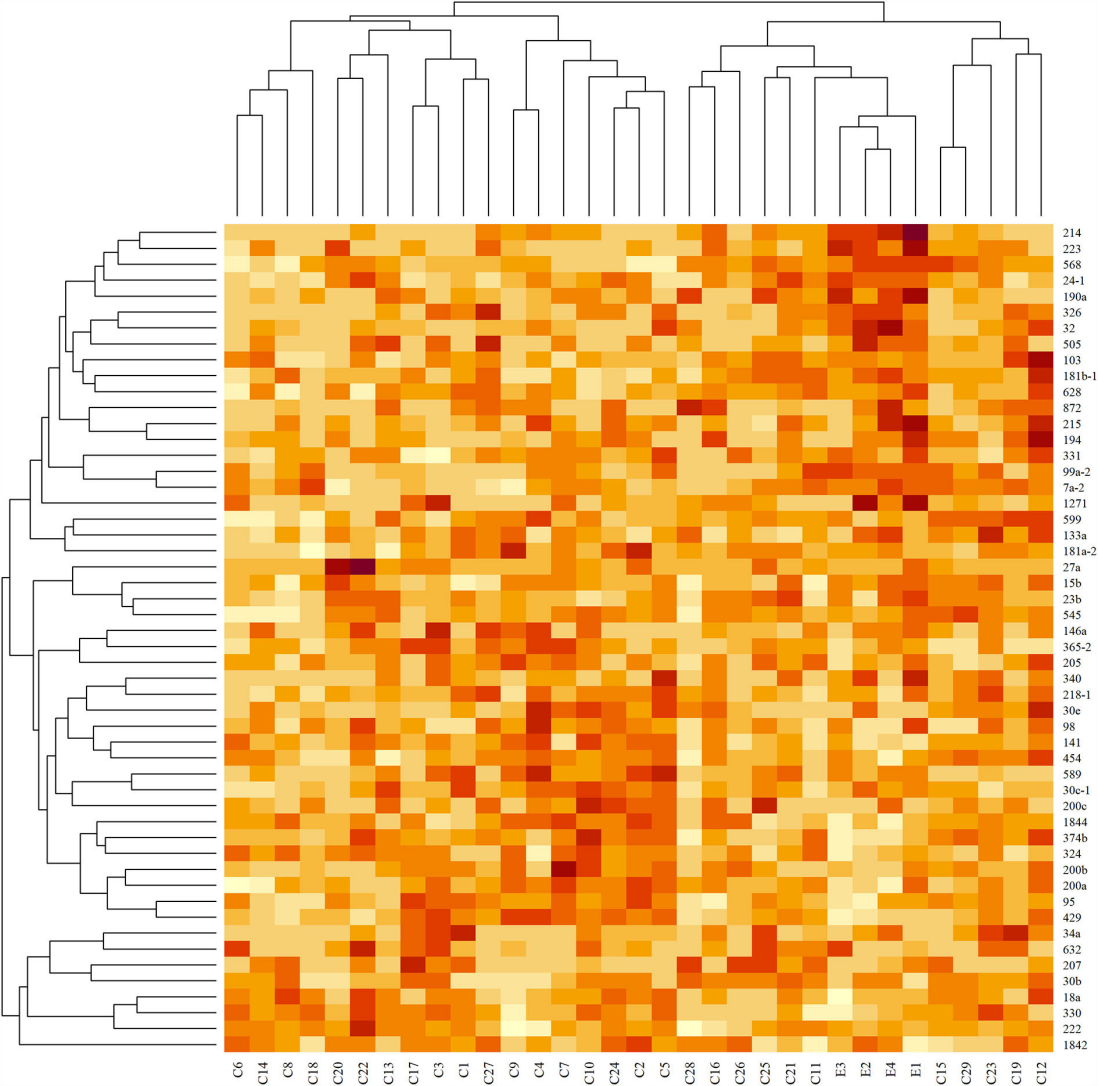
Heatmap of standardized read counts for filtered miRNA subset. Darker colors correspond to higher read counts. Dendrograms were derived with agglomerative hierarchical clustering with Pearson's correlation distance and average linkage. Tissue samples are labeled along the x-axis, and miRNA samples are labeled along the y-axis. Increased expression of miR-214, miR-223, miR-568, miR-24-1, miR-2901, and miR-32 are seen in the control samples (E) compared to invasive urothelial carcinoma samples (C).

**Figure 3:**
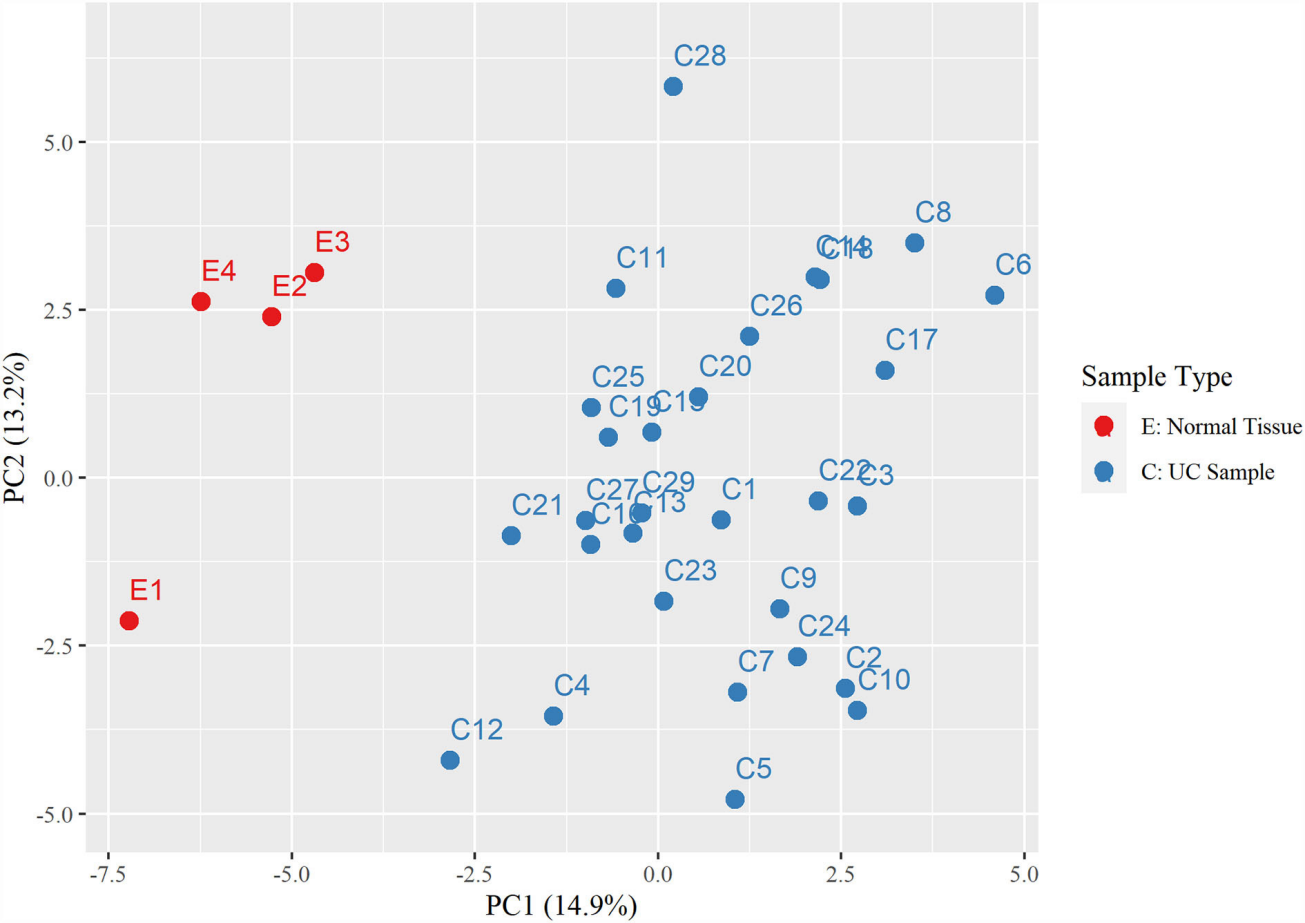
Principal component analysis displaying a distinct separation between the control urothelial tissues (E1-4) and the invasive urothelial carcinoma samples (C1-29).

Hierarchical clustering based on filtered miRNA subset demonstrates mutual similarity of the normal tissue samples relative to the iUC samples (Figure 1). A heatmap of the Pearson's correlation distance measure compared to miRNA expression highlighted increased expression of miR-214, miR-223, miR-568, miR-24-1, miR-190a, and miR-32 (Figure 2). Principal component analysis based on filtered miRNA subset showed that normal tissue and iUC samples are clearly separated from the first principal component, with low overall variance (14.9%) (Figure 3). No distinction was found when differentiating tumor subtype (luminal or basal), immune score (score 1-5 from lowest non-T-cell inflamed to highest T-cell inflamed) (41), the presence of the BRAF mutation, or tumor grade (tumor grades 2-4 included in this study) (41) (Supplementary Figures 1, 2).

### Reverse-transcription quantitative PCR

#### Reference gene identification

Both miR-1842 and RNU6B were acceptable for use as reference genes for miRNA expression using FFPE tissues, according to NormFinder (Table 2), and used in combination to increase the accuracy of the normalization (42).

#### RNA-Seq validation

Four of the five miRNAs evaluated (miR-105a, miR-143, miR-181a, and miR-214) were significantly downregulated (*P* < 0.05, FC < 2) in canine iUC compared with normal control urothelium (Table 3). Although there was a trend toward downregulation, miR-32 was not statistically DE.

### Pathway analysis

**Figure 4:**
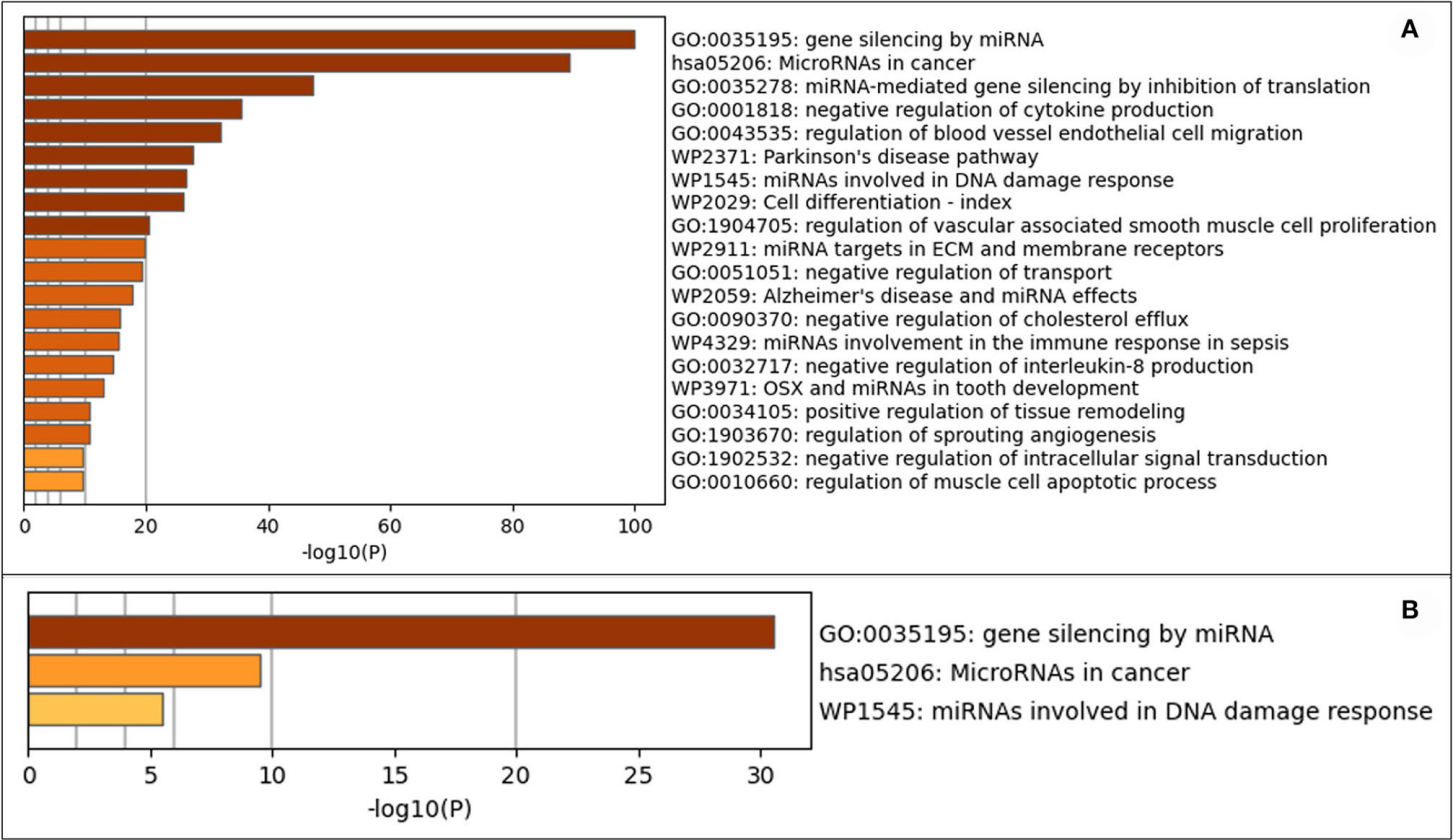
Gene annotation analysis for statistically enriched terms using Mtascape. **(A)** All expressed miRNAs. **(B)** Differentially expressed miRNAs found in invasive urothelial carcinoma samples compared to normal urothelial control tissues.

**Figure 5:**
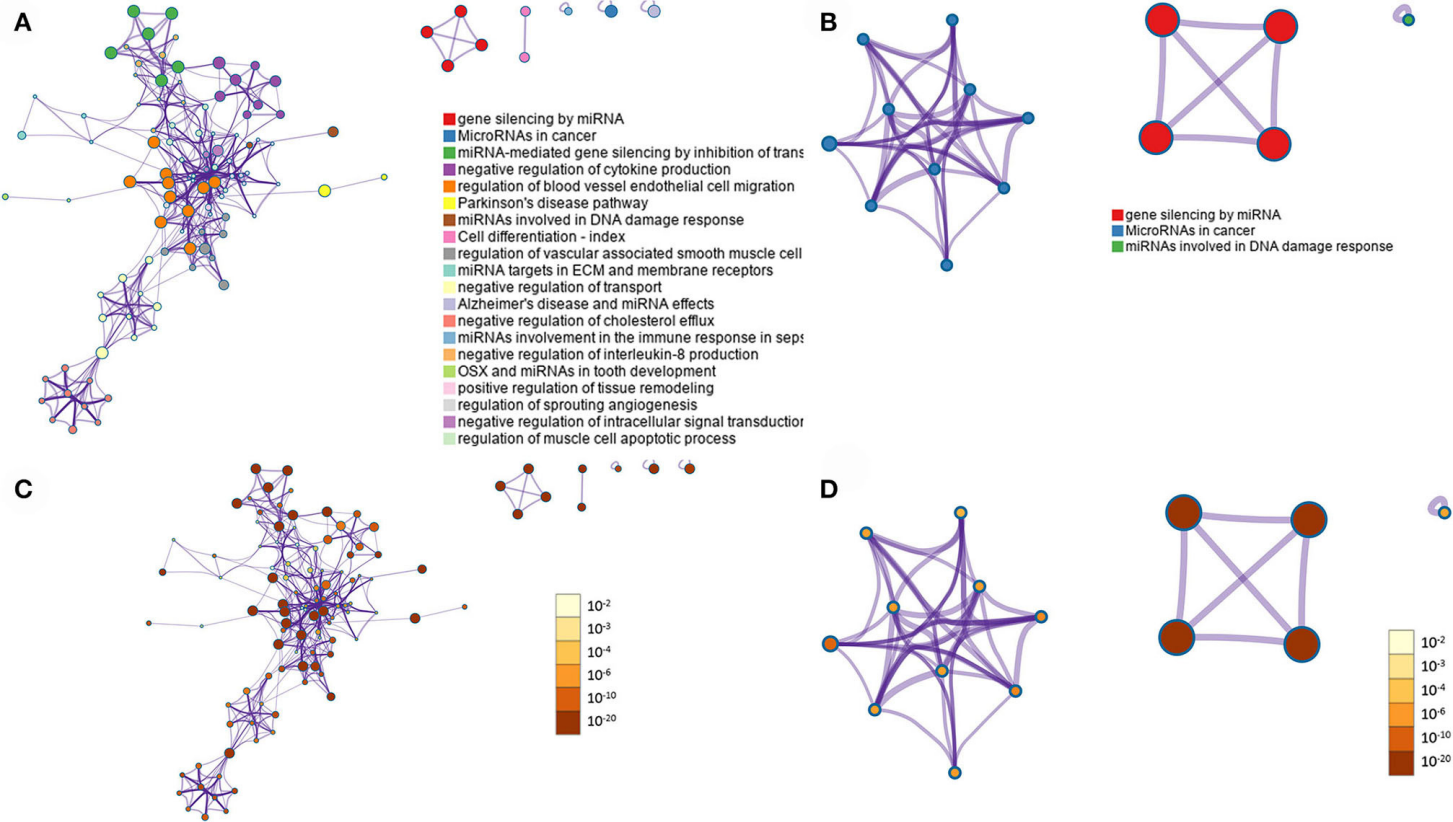
Summary of enrichment analysis. Network analysis of the GO terms with the different colored nodes representing the terms within the pathways. **(A)** All miRNAs expressed. **(B)** Differentially expressed miRNAs. Nodes are also evaluated by p-value, with the darker colors representing lower p-values, indicating higher significance of the node. **(C)** All miRNAs expressed. **(D)** Differentially expressed miRNAs. Images prepared by Metascape.

Metascape software was used to evaluate the 208 expressed miRNA (Supplementary Table 5) and the 28 DE miRNAs (Supplementary Table 6). Metascape uses humans as the target organism for this evaluation due to the similarity of the molecular behavior of canine and human iUC (1, 3–5); an assumption was made that similar pathways would be enriched in dogs as are found in humans. When evaluating all expressed miRNAs, the top three out of 100 associated terms were: gene silencing by miRNA, miRNAs in cancer, and miRNA-mediated gene silencing by inhibiting translation (Figure 4A). The evaluation of only the DE miRNAs showed only the terms: gene silencing by miRNA, miRNAs in cancer, and miRNAs involved in DNA damage response (Figure 4B). The network pathway of these terms shows six distinct networks in all expressed miRNA (Figure 5A) and three distinct networks in the DE miRNAs (Figure 5B). The network nodes were also classified by p-value in the expressed miRNAs (Figure 5C) and the DE miRNAs (Figure 5D).

**Figure 6:**
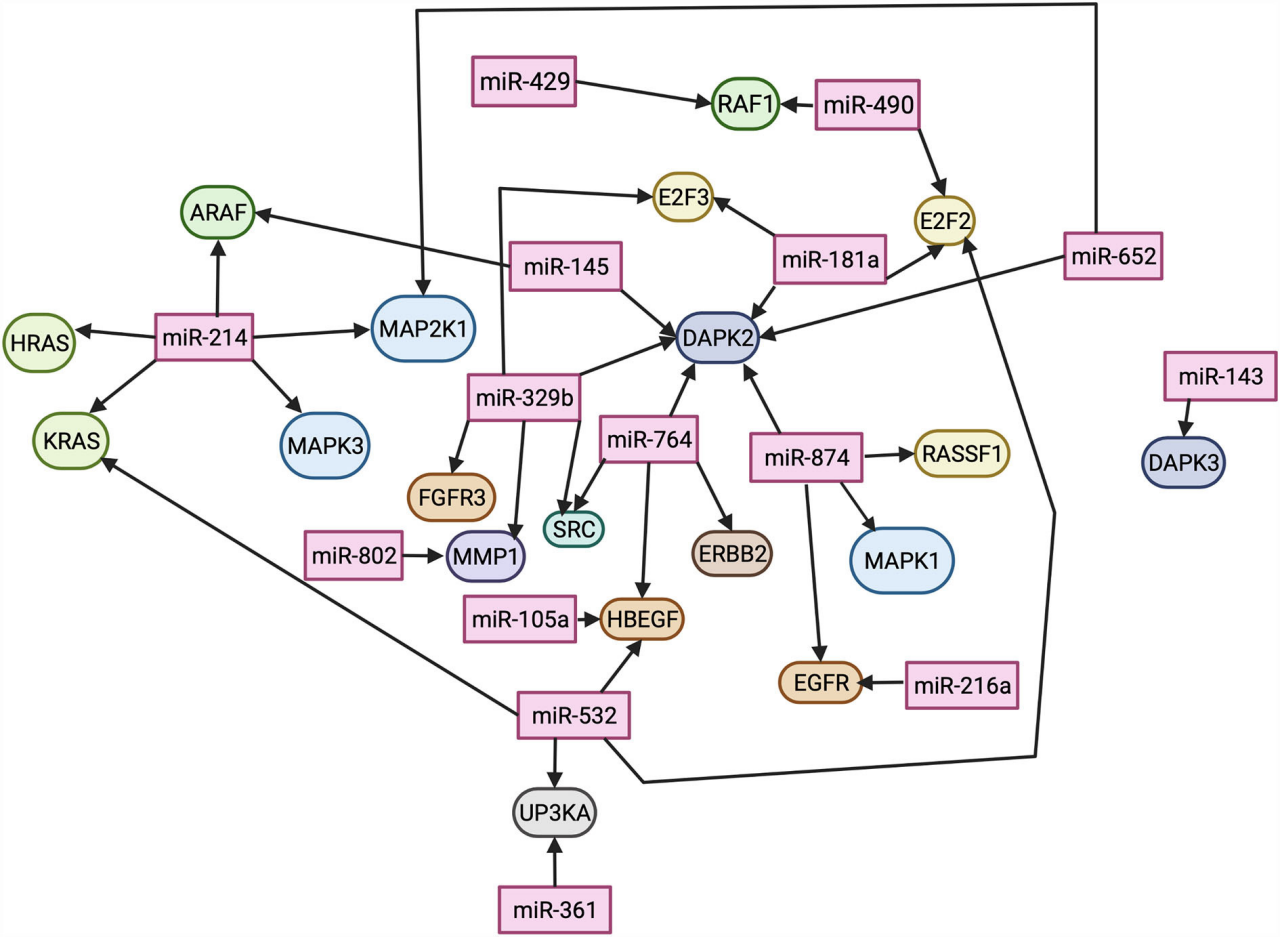
Differentially expressed miRNAs and their predicted protein targets, evaluated through miRWalk. Lines connecting miRNAs to proteins indicate an interaction. Image created using BioRender.

Twenty-nine pathways were found enriched: *EGF, MDM2, ERBB2, MAP2K1, MAPK1, DAPK1, MAPK3, NRAS, HRAS, CDH1, TP53, EGFR, RAF1, RASSF1, DAPK3, E2F1, SRC, KRAS, THBS1, DAPK2, E2F3, ARAF, HBEGF, MMP2, E2F2, FGFR3, MMP1, RPS6KA5*, and *UPK3A*. Network analysis for these pathways was further performed through STRING for the *Canis lupus familiaris* database (Supplementary Table 7). The DE miRNAs were evaluated for protein pathway associations using the miRWalk database (Figure 6).

Thirteen DE miRNAs (miR-582, miR-991-2, miR-544, miR-568, miR-450a, miR-301a, miR-876, miR-32, miR-7-3, miR-190a, miR-551b, miR-223, and miR-374b) were not previously reported nor had predicted pathways associated with iUC in dogs. Of these, five (miR-582, miR-99a-2, miR-544, miR-568, miR-450a) had no GO term association. Metascape term enrichment analysis found an association with gene silencing by miRNA (GO: 0035195), post-transcriptional gene silencing by RNA (GO: 0035194), and post-transcriptional gene silencing (GO: 0016441) for five of these miRNAs (miR-876, miR-7-3, miR-901, miR-551b, and miR-374b) in the human database. Additionally, Metascape enrichment analysis within the human literature predicted that miR-301a was associated with positive regulation of vascular associated smooth muscle cell proliferation (GO:1904707), regulation of vascular associated smooth muscle cell proliferation (GO:1904705), and positive regulation of smooth muscle cell proliferation (GO:0048661); miR-32 was associated with Wnt signaling pathway (GO:0016055), cell-cell signaling by Wnt (GO:0198738), and cell surface receptor signaling pathway involved in cell-cell signaling (GO:1905114); miR-223 was associated with negative regulation of granulocyte chemotaxis (GO:0071623), negative regulation of neutrophil chemotaxis (GO:0090024), negative regulation of neutrophil migration (GO:1902623).

## Discussion

In this study, we have described the miRNome of iUC using RNA-Seq and validated the data by RT-qPCR. The RNA-Seq analysis revealed that 208 miRNAs were expressed in the urothelial tissue with only 28 DE miRNAs (differentially expressed between normal mucosa and iUC). Of the 28 DE miRNAs, 15 were predicted to have associations with pathways known to be altered in canine UC. The validation by RT-qPCR was performed in a different cohort of archived FFPE samples. Four miRNAs were confirmed to be downregulated in iUC compared to normal urothelium from the five miRNAs evaluated. Although a trend toward downregulation was seen, miR-32 did not show significant differential expression, likely due to the limited sample size. Nonetheless, independent validation combined with the pathway analysis shows that the data generated by RNA-Seq is biologically significant. The most robust pathways identified by STRING analysis included the RAS (from “rat sarcoma virus”) oncogenic pathway, the regulation of growth factors including epithelial growth factor receptor (EGFR) and fibroblast growth factor receptors (FGFRs), the danger activated protein kinase (DAPK) pathway, uroplakin protein IIIa (UPK3A), and the E2 factor (E2F) family of transcription factors pathways.

The RAS pathway regulates many cellular processes such as differentiation, proliferation, motility, and transformation (43); It is one of the first described oncogenic pathways and the most altered in human cancers. RAS then activates the mitogen-activated protein kinase (MAPK) pathway in the BRAF/MEK/MAPK signaling axis, resulting in the upregulation of transcription factors that support cell proliferation and survival (44). These pathways are regulated by many post-transcriptional factors, including miRNAs (44). Herein, we identified the downregulation of miR-145, miR-214, and miR-532 in iUC, which are associated with RAS. MiR-145 and miR-214 are experimentally confirmed to target RAS expression; miR-145 is a tumor suppressor found downregulated in several cancers and associated with cancer progression by targeting RAS (45). MiR-145 is also part of the p53 and c-Myc regulatory network, and its downregulation is associated with cancer initiation and development through this pathway. Notably, miR-532 is downregulated in renal cancer through its role in the MAPK pathway (46), and its downregulation results in the proliferation and invasion of bladder cancer in humans through the activation of the Wnt/β-catenin signaling pathway (47). Additionally, our study identified three downregulated miRNAs associated with MAPK regulation by STRING analysis: miR-214, miR-652, and miR-874. MiR-214 induces the MAPK signaling pathway activity increasing cell proliferation and inhibiting apoptosis (48). While miR-652 is downregulated herein and in many cancers; however, it is found upregulated in human bladder cancer (49), adding to the studies reporting different molecular mechanisms of urothelial carcinoma in dogs and humans (50). Similarly, miR-874 was not DE in a study of the human miRNome and was used as a reference gene in that study (29). Comparative studies are warranted to elucidate miRNA regulation of UC in dogs and humans, as many research groups suggest using the dog as a natural animal model of disease (4). Nevertheless, our study suggests that the downregulation of miRNAs associated with the RAS/MAPK pathways may play a role in canine iUC tumorigenesis (23, 51–53).

It is noteworthy that BRAF is part of the RAS pathway as an activator of the MAPK (43). The BRAF mutation, identified in ~80% of canine UC cases (8, 10), results in inappropriate activation of the *MAPK* pathway with consequent abnormal proliferation and differentiation of cancers, including human and canine UC (43, 54). Interestingly, experimental data in human cancers strongly suggest that dysregulated miRNA expression can activate RAS independently of their oncogenic mutations (54). However, it is not clear if there is a direct association between the altered miRNAs associated with those pathways and the presence of the mutation in this study, as no clear sample separation was evidenced by PCA analysis when analyzing the presence of BRAF mutation in the RNA-Seq data, likely because most iUC samples (25/29) had the BRAF mutation. Nevertheless, additional experimental studies must be conducted to explore those associations.

Growth factors have been extensively researched in cancers, including bladder cancer, because of their role in cancer biology and clinical relevance as prognostic and therapeutic targets, and EGFR is a well-characterized initiator of the RAS signaling pathway upon activation by EGF (30). Both EGFs and FGFRs have reported prognostic and therapeutic value in human UC. Abnormal expression of the EGFR pathway was associated with muscle-invasive bladder cancer and indicated a poor prognosis, and FGFR3 mutations or overexpression indicated a more favorable prognosis (55, 56). Moreover, anti-EGFR treatment has shown promise in treating metastatic urothelial carcinoma in humans (57), as well as antitumor activity in canine iUC (58). Herein we found downregulation of miRNAs predicted to regulate EGF (miR-105a, miR-216a, miR-532, miR-874) and FGFR (miR-329b). These miRNAs have been shown to have numerous associations with various other cancers (59–65). Furthermore, another miRNA (miR-764) predicted to regulate the growth factor ERBB (erythroblastic leukemia viral oncogene homolog), also known as *HER*, was downregulated in iUC compared to controls. ERBB belongs to a family of proteins containing receptor tyrosine kinases related to the EGFR, and this interaction promotes the development of UC. ERBB functions as a receptor for a tyrosine kinase that can directly affect the RAS signaling pathway (66). ERBB has been associated with other neoplasms; of most note is its association with breast cancer (66). In canine UC, ERBB has been found to be expressed most frequently in the luminal subtype of UC, and the luminal subtype has been associated with a better prognosis than the basal subtype (5, 41). Although, in this study, no distinguishable separation was found between miRNAs in the iUC subtypes on PCA (Supplementary Figure 2). Downregulation of these regulatory miRNAs will result in overexpression of their target genes. Given the role of these growth factors in cancer development and consequent use as prognostic and therapeutic markers, it is plausible to suggest that their correspondent regulatory miRNAs are also potential targets and should be further investigated. Several studies in animal models have shown that downregulated miRNAs that act as tumor-suppressors can be restored and potentially aid in cancer therapy (67).

MiRNA-361 is reported to regulate epithelial to mesenchymal transition through the extracellular signaling-related kinases (ERK), signaled by RAS(68), and acts as a tumor suppressor in prostate cancer by targeting signal transducer and activator of transcription-6 (STAT6), a pro-apoptotic signaler to BCL-xL (69). This study reports two downregulated miRNAs, miR-532 and miR-361, which were predicted to regulate UPK3A expression. Uroplakins are membrane proteins that terminally differentiate to create specialized areas of urothelial membrane called urothelial plaques, which give the bladder its flexibility and the ability to be impenetrable (70). MiRNAs have been previously reported to affect the development of uroplakins by targeting the tight junction-related proteins during the differentiation of urothelial cells (71). Interestingly, overexpression of UPK3A is well-documented in urothelial carcinomas, and UPK3A is widely used as a marker of UC by immunohistochemistry in humans and dogs (6, 72).

Death-associated protein kinases (DAPK) are a family of proteins that regulate apoptosis and non-apoptotic cell death (73). Overexpression of members of the DAPK family (DAPK, Zipk, and DRP-1) is known to cause cell death (73). DAPK has been shown to encode calcium/calmodulin-regulated serine/threonine kinase to induce apoptosis and suppress tumor growth (74). The influx of calcium induces phosphorylation and activation of DAPK, and activation of Beclin-1, inducing phagophore formation (75). This method of tumor suppression has been documented in human bladder cancer (74, 76), and methylation of DAPK has been suggested as a diagnostic marker for bladder cancer in humans (73, 74, 76, 77). In addition, DAPK is one of the pro-apoptotic genes activated by p53 (75). Many DE downregulated miRNAs in this study were found to affect the DAPK family of proteins: miR-145, miR-181a, miR-329b, miR-764, miR-874, miR-652, and miR-143. Interestingly, upon literature review, only miR-103 and miR-107 have been shown to target DAPK, promoting the development of colorectal cancer (75). The findings of this study suggest that there are numerous previously unreported miRNAs that may suppress DAPK and contribute to canine iUC development.

Our study also identified miRNAs involved in regulating the transcription factors E2F2 and E2F3. The E2F family of transcription factors has been extensively studied in the context of cancer development; they play a crucial role in cell cycle progression preferentially through the retinoblastoma protein p53 pathways. It is also well-established that E2F transcription factors are regulated by miRNAs. On the other hand, these factors can regulate miRNA expression through negative feedback in a complex regulatory network (78). Altered expression of these factors has been reported in UC and prostatic carcinoma (79). Three miRNAs with predicted E2F2 association were DE: miR-490 was upregulated and miR-181a and miR-532 were downregulated, whereas two miRNAs, miR-329b and miR-181a, associated with E2F3 were downregulated. Increased expression of E2F3 has been found with advanced tumor stage of human bladder cancer (79). Experimental studies linking these miRNAs with the E2F family are lacking; our study serves as base information for further investigations on these interactions.

Thirteen miRNAs with no reported canine UC pathway association were DE in this study and represent an opportunity for further evaluation. While in the human literature, five of these miRNAs (miR-876, miR-7-3, miR-901, miR-551b, and miR-374b) are involved in gene silencing, known to be a function of miRNAs and a contributing factor to cancer development, three of these have associations with vascular development, cell signaling, and negative chemotaxis regulation (miR-301a, miR-32, and miR-223, respectively) (80–82). MiR-301a is also reported to downregulate p63, resulting in decreased expression of E-cadherin, which is associated with increased potential for prostate cancer reoccurrence (83). MiR-301a is also associated with increased cell proliferation and invasion in colorectal cancer by targeting RUNX family transcription factor 3 (84) and targeting of suppressor of cytokine signaling 6 (85). In pancreatic cancer, miR-301a acts as an activator of the nuclease factor kappa beta (86), associated with numerous cell processes such as cell tumorigenesis, apoptosis, immunity, and inflammation. MiR-32 targets PTEN, a tumor suppressor that inhibits angiogenesis and cell proliferation (87, 88). MiR-223 has numerous previously reported targets and associations with several cancers and inflammatory conditions (89). Our findings indicate that numerous miRNAs and pathways are understudied that may contribute to the development of canine iUC.

A limitation of this study was that different breeds were used for both iUC and control samples. While this may be representative of the general canine population presenting with iUC and the histologic appearance and behavior of iUC in dogs are similar regardless of the breed (4, 5, 41, 90), the effects of the difference in the genetic source are not evaluated in this study. Additionally, although the genes discussed in this study, EGFR, MDM2, ERBB2, MAPK1, HRAS, CDH1, TP53, RASSF1, THBS1, HBEGF, FGFR3, MMP1, UPK3A, and MAP2K1, were also dysregulated in the transcriptome (5), a direct connection of miRNA and mRNA regulation cannot be made as miRNAs are known to have multiple gene targets (13). Data integration and miRNA functional studies in canine iUC are warranted.

In conclusion, this study has characterized the miRNome in canine iUC. We describe 15 DE miRNAs associated with 29 known protein pathways altered in canine iUC, 13 DE miRNAs not associated with known canine iUC pathways, and three associated with pathways not predicted to be altered in iUC. Although the potential of miRNA use as diagnostic markers, prognostic markers, and therapeutic targets has been recognized (91), the paucity of studies on canine diseases and samples indicates a need for additional investigation. This study highlights numerous miRNAs and miRNA targets, supporting previously reported BRAF/MEK/MAPK signaling pathway and newly identified DAPK signaling, UPK3A, and E2F transcription factors that advance the understanding of miRNA interactions and canine iUC development. The discovery of multiple altered miRNAs in different pathways is noteworthy since they can be further investigated as diagnostic markers of iUC, especially for cases that do not harbor common mutations.

## Data availability statement

The datasets presented in this study can be found in online repositories. The names of the repository/repositories and accession number(s) can be found in the article/Supplementary materials.

## Ethics statement

The animal study was reviewed and approved by Purdue Animal Care and Use Committee (Approval Number 1111000169). Written informed consent was obtained from the owners for the participation of their animals in this study.

## Author contributions

Conceptualization: AS, MV, DD, and DK. Data curation: AS, DD, and MV. Formal analysis: DD, TB, and MV. Funding acquisition: AS and DK. Investigation: MV, AS, DD, JR-V, and DK. Methodology and visualization: MV, AS, and DD. Resources: AS, JR-V, and DK. Supervision: AS, DD, and DK. Validation, project administration, and writing—original draft: MV and AS. Writing—review and editing: MV, TB, DD, DK, JR-V, and AS. All authors contributed to the article and approved the submitted version.

## Funding

Funding for RNA-Seq evaluation was provided by the National Institutes of Health/National Cancer Institute grant P30CA023168 Supp (DK). The funders had no role in study design, data collection, or analysis. The Purdue University Andrews Fellowship provided salary support for MV. Additional funding was provided by AS' laboratory.

## Conflict of interest

The authors declare that the research was conducted in the absence of any commercial or financial relationships that could be construed as a potential conflict of interest.

## Publisher's note

All claims expressed in this article are solely those of the authors and do not necessarily represent those of their affiliated organizations, or those of the publisher, the editors and the reviewers. Any product that may be evaluated in this article, or claim that may be made by its manufacturer, is not guaranteed or endorsed by the publisher.
