# Supplementary material for: The miRNome of canine invasive urothelial carcinoma
Source: Front Vet Sci. 2022 Aug 22;9:945638. doi: 10.3389/fvets.2022.945638 (PMC9443663; doi:10.3389/fvets.2022.945638)
Supplement: Supplementary file 6 [file Data_Sheet_1.DOCX]

Supplementary Material

# Supplementary Data

Supplementary Material should be uploaded separately on submission. Please include any supplementary data, figures and/or tables. All supplementary files are deposited to FigShare for permanent storage and receive a DOI.

Supplementary material is not typeset so please ensure that all information is clearly presented, the appropriate caption is included in the file and not in the manuscript, and that the style conforms to the rest of the article. To avoid discrepancies between the published article and the supplementary material, please do not add the title, author list, affiliations or correspondence in the supplementary files.

# Supplementary Figures and Tables

**Supplementary Table 1.** Tumor classifications for each patient with iUC.

**Supplementary Table 2.** TMM and DESeq normalized RNA-Seq data depicting miRNAs expressed in canine bladder urothelial tissue.

**Supplementary Table 3.** Gene Set Enrichment Analysis **(**GSEA) analysis for bladder cancer in dogs.

**Supplementary Table 4.** Depicts the numbers of miRNAs expressed in only control canine urothelial tissue, only iUC, in both in normal control canine urothelial tissue, and miRNA that are minimally expressed.

**Supplementary Table 5.** Metascape prepared enrichment analysis for all expressed miRNAs.

**Supplementary Table 6.** Metascape prepared enrichment analysis for DE miRNAs.

**Supplementary Table 7.** String database prepared GO term enrichment for Gene Set Enrichment Analysis (GSEA) identified protein pathways.

## Supplementary Figures

**Supplementary Figure 1.** Principal component analysis based on filtered miRNA subset stratified by descriptive characteristics of subtype and immune score. A. Samples colored by tumor subtype. There appears to be a moderate level of distinction between basal and luminal subtypes when accounting for the normal tissues. However, the two subtypes are indistinguishable when normal tissues are removed from the analysis. B. Samples colored by Immune score. No apparent trend is identified.

**Supplementary Figure 2.** Principal component analysis based on filtered miRNA subset stratified by descriptive characteristics of BRAF mutant expression and Tumor grade. A. Samples colored be BRAF genotype. No apparent trend is identified. Highly unbalanced subsample size is noted.
B. Samples color by tumor grade. No apparent trend identified.
