# Supplementary figures and images for: The miRNome of canine invasive urothelial carcinoma
Source: Front Vet Sci. 2022 Aug 22;9:945638. doi: 10.3389/fvets.2022.945638 (PMC9443663; doi:10.3389/fvets.2022.945638)

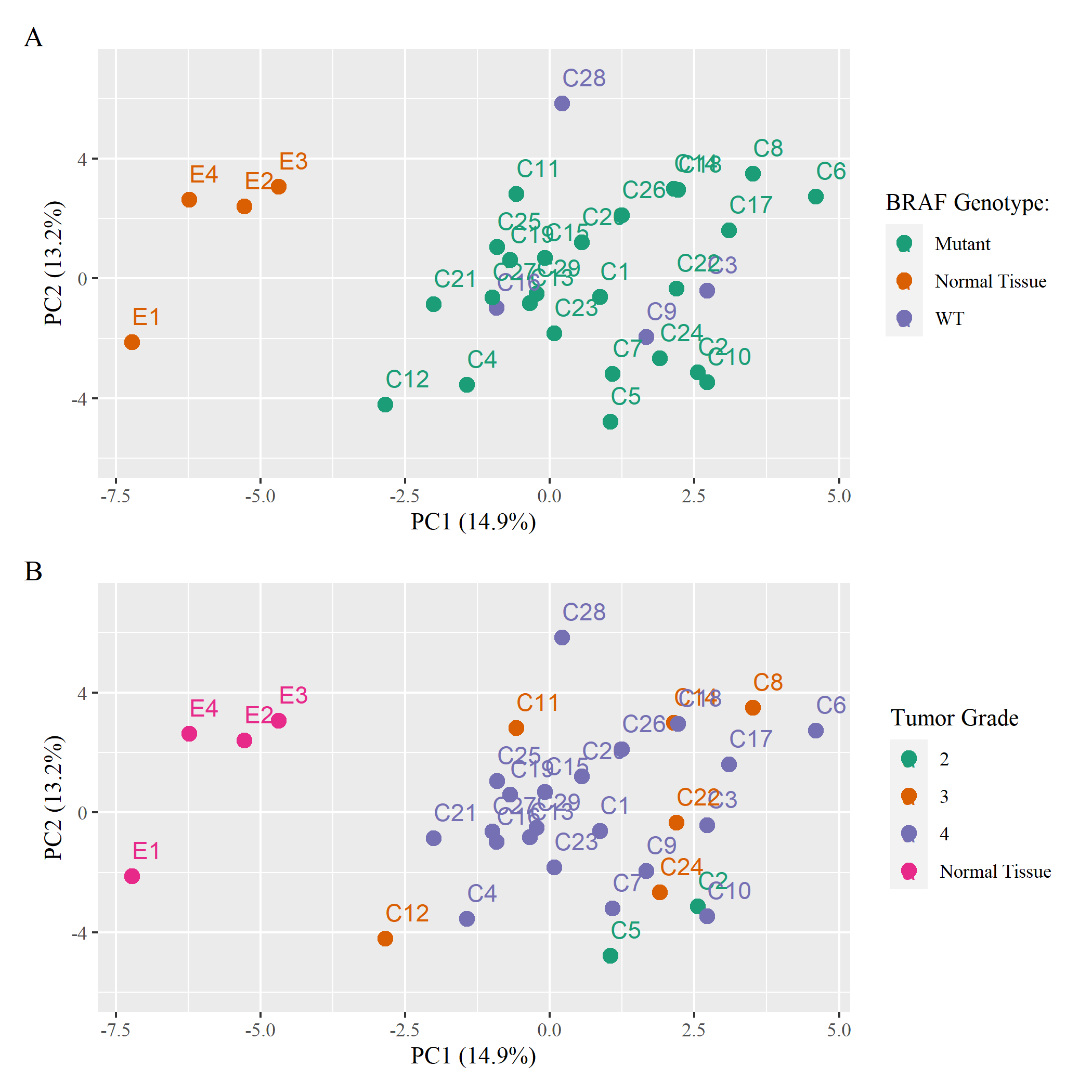

Supplement: Supplementary file 9 [file Image_1.TIFF]

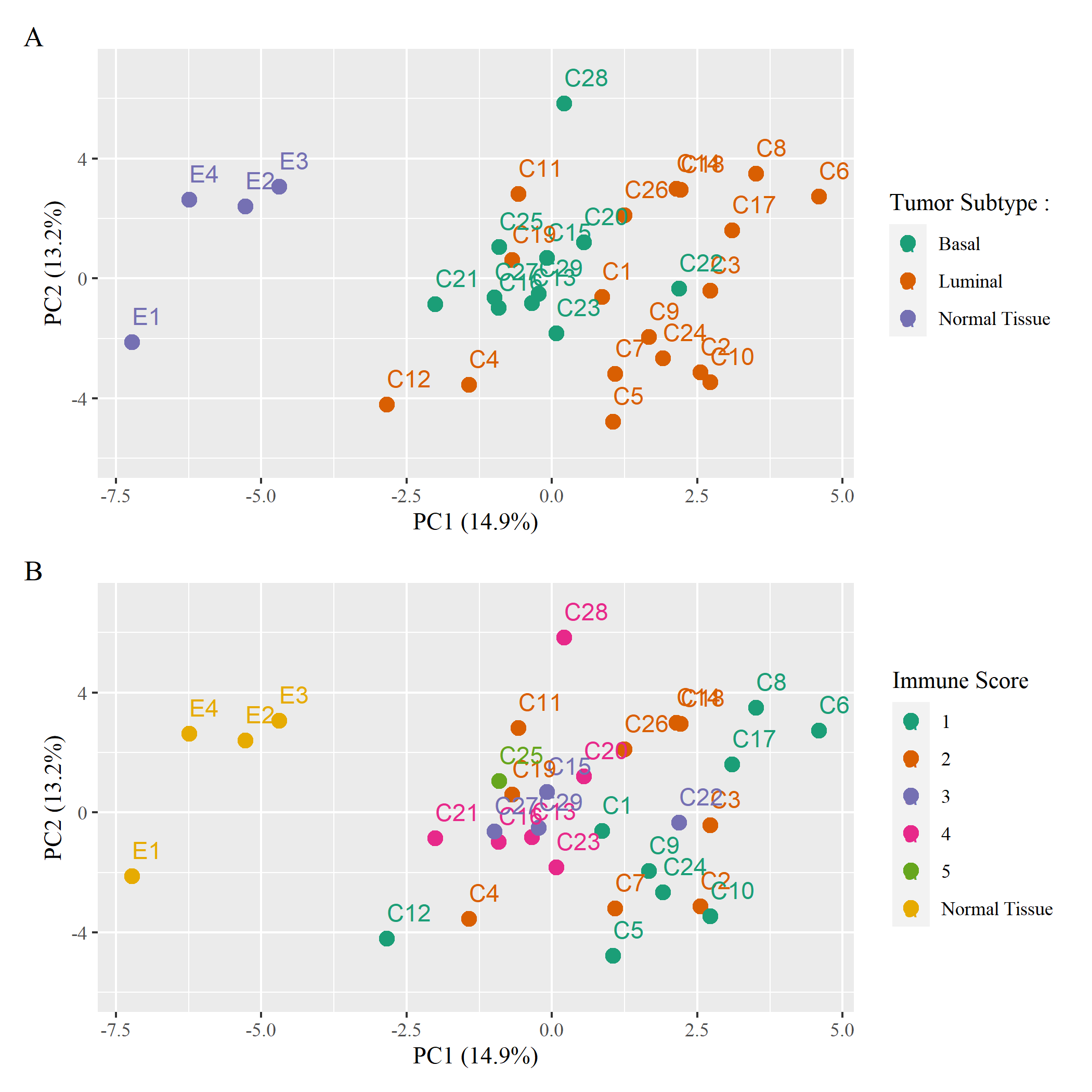

Supplement: Supplementary file 10 [file Image_2.TIFF]
